# Body Mass Index of Male Youths Aged 18-20 Years of the Han Nationality Living in Different Regions of China

**Published:** 2007-12

**Authors:** Lei Shang, Xun Jiang, Xiang-hong Bao, Fu-bo Xue, Yong-yong Xu

**Affiliations:** 1 Department of Health Statistics, Faculty of Preventive Medicine, Fourth Military Medical University, Xi'an 710032, Shaanxi, China; 2 Department of Pediatrics, Tangdu Hospital, Fourth Military Medical University, Xi'an 710038, Shaanxi, China

**Keywords:** Adolescent, Anthropometry, Body mass index, Nutritional status, Obesity, China

## Abstract

The study was conducted to assess the nutritional status and levels of body mass index (BMI, kg/m^2^) and to evaluate the geographical distribution of male youths of the Han nationality in China. In total, 60,773 male youths, aged 18-20 years, of the Han nationality, were categorized into underweight, normal-weight, overweight, and obesity according to the international adult BMI cut-offs. Different levels of nutritional status and BMI of male youths of the Han nationality were compared among different areas. The mean BMI for the whole country was 20.6 in urban areas and 20.0 in rural areas. BMI increased from 20.1 among 18-year old youths to 20.5 among 20-year old youths. The prevalence of underweight among the male youths was 21.6%, while the prevalence of overweight and obesity were 4.6% and 0.6% respectively. For urban youths, the prevalence of underweight, overweight, and obesity were 21.0%, 6.8%, and 1.1% respectively, while these were, respectively, 21.9%, 3.3%, and 0.3% for rural youths. The nutritional status of the male youths in North-China was at the highest level (21.1) among the six areas, and the prevalence of underweight, overweight, and obesity were 14.3%, 9.1%, and 1.4% respectively. The highest prevalence of underweight was 29.8% in the North-West region, and the lowest prevalence of overweight was 2.2% in the South-Middle region, while the lowest prevalence of obesity was 0.2% in the South-West region. The nutritional status of the male youths was significantly different among different areas. Underweight was still prevalent in all male youth groups. Nonetheless, overweight was more prevalent among urban youths than among rural youths and was more prevalent in the North region than in the South region.

## INTRODUCTION

Nutritional status may be related to the health status of the individual because of its association with the risks and consequences of diseases (1–7); for example, overweight and obesity are important determinants of the onset of adult diabetes and heart diseases (1). Youth is a growth period between childhood and adulthood; so, the nutritional status of youths can not only reflect the physique status of lifestyle of one's early life, but also foretell one's physique status in later life. Once they are 18 years old, youths are required to make a living independently, and after a few more years, they are required to be responsible economically for their families; health is, thus, crucial for a male youth himself, his family, and the entire society. It is, therefore, important to investigate the epidemiological characteristics of the nutritional status of male youths and its influencing factors to decrease the prevalence of malnutrition (underweight and obesity). This study was undertaken to measure body mass index (BMI, i.e. weight/height^2^, kg/m^2^) and nutritional status of male youths of the Han nationality to compare its geographical distribution characteristics. Thus, it can provide scientific evidence for administrative wings of the health departments at all levels to devise appropriate health policies and health-education plans to improve the nutritional status of male youths in China.

## MATERIALS AND METHODS

### Subjects

The research group conducted a national survey on physical status and common diseases of youths recruited for the Chinese military in 2001. One hundred survey-spots were selected by the cluster-sampling method among 2,861 counties or cantons in China. The sampling was restricted to 27 provinces or autonomous regions and four municipalities, except Hong Kong, Taiwan, and Macao. According to the population, geography, and economy of each selected province, 2-4 counties or cantons were designated as sampling-spots. In each survey-spot, all recruited youths were surveyed, and data on 81,193 recruited youths were collected.

According to the data, female youths, the minority subjects, male youths aged below 18 years or over 20 years, and subjects with chronic gastroenteritic diseases, endocrinopathy, or other diseases which could cause abnormal reduction or gaining in weight were all rejected. Thus, the sample included 60,773 subjects for analysis.

Measurement of BMI and nutritional status was conducted in two weeks in November 2001 from 8 am to 12 noon each day.

### Criteria of diagnoses

The approach focused on the measurement of current status of underweight and overweight. Using internationally-accepted BMI cut-offs recommended for an adult by the World Health Organization (WHO), the research defined BMI <18.5 as underweight, BMI 18.5∼25 as normal-weight, BMI 25∼30.0 as overweight, and BMI ≥30 as obesity (7).

### Anthropometric indexes

Height and weight were measured following standard methods (8), and the same precision instruments were used by each task group. The observers for each item were all trained before measurements in the field.

### Height

A metal column height-metre (200 cm long with 0.1 cm precision) was used for measuring height. The subjects were required to stand barefooted and with their arms straight and relaxed by their sides on the measuring instrument. The heels, buttock, and scapulars were in contact with the column of the height-metre, and the heads were held in the Frankfort plane when the distance from the top of the scales to the feet was being measured.

### Weight

For measurement of weight, beam scales (each weighing 120 kg with 0.1 kg precision) were used. The subjects were required to wear only shorts when the measurement took place. During the measurement period, the observer checked the instrument every day to ensure that the scales were accurate and sensitive.

### Areas investigated

China is divided into six areas: North-East area, including Liaoning province, Jilin province, Heilongjiang province, and Inner Mongolia autonomous region; North-China area, including Beijing city, Tianjin city, Shanxi province, Shandong province, Hebei province, and Henan province; East-China area, including Shanghai city, Jiangsu province, Zhejiang province, Anhui province, Jiangxi province, and Fujian province; South-Middle area, including Hubei province, Hunan province, Guangdong province, Guangxi Chuang autonomous region, and Hainan province; North-West area, including Shaanxi province, Gansu province, Ningxia Hui autonomous region, Xinjiang Uighur autonomous region, and Qinhai province; South-West area, including Sichuan province, Guizhou province, Yunnan province, Chongqing city, and Tibet autonomous region.

The research group recruited subjects at the national level and in every administrative region. They provided technical training, conducted measurements in the field, and collected data. The survey staff included medical researchers, physicians, nurses, and technicians who were trained together before the survey. In each survey-spot, each member was assigned the responsibility to measure or examine a specific item.

### Statistics

The database was built with the Epi Info software (version 2000), a set of programs for word processing, data management, and epidemiologic analysis, designed for public-health professionals recommended by WHO for free use. Since the proportion of age of subjects significantly diferred among different areas, we adjusted the proportion of age of subjects for each area according to the age proportion of the whole subjects: 48.77% of the subjects were aged 18 years, 30.03% were aged 19 years, and 21.19% were aged 20 years. Under this presupposition, the Kruskal-Wallis H-test and the Mann-Whitney U-test were performed. The statistical differences in nutritional status among different areas were compared. All statistical analyses were carried using the SPSS software (version 13.0).

## RESULTS

### BMI of male youths of the Han nationality in different areas

Table 1 shows the comparison of BMI in male youths aged 18-20 years in different areas. The table shows that the differences in BMI among various groups were not only reflected in different age-groups of the same area, but also in different geographical areas. The mean BMI for the whole country was 20.6 in urban areas and 20.0 in rural areas. It increased from 20.1 for 18-year old youths to 20.5 for 20-year old youths as the age increased. BMI of male youths in North-China was the highest, which was 21.1 for the whole region, with 20.9 for 18-year, 21.2 for 19-year, and 21.6 for 20-year old youths, and with 21.6 in urban areas and 20.9 in rural areas. There were a significant difference (p<0.05) in BMI among different age-groups in the same area. For the same age-group, there were significant differences in BMI among different areas, except in the South-Middle and West-North, West-North and West-South, and South-Middle and West-South areas.

**Table 1:** Comparison of BMI (kg/m^2^) in male youths aged 18-20 years in different areas of China∗

		Urban areas			Rural areas			Total			
Area	Age(years)	No.	Mean	95% CI	No.	Mean	95% CI	No.	Mean	95% CI

North-East	18	1,350	20.9†	20.8-21.1	1,160	20.3	20.1-20.4	2,510	20.6	20.5-20.7
	19	871	21.2†	21.0-21.4	590	20.7	20.5-20.9	1,461	21.0	20.9-21.2
	20	528	21.1†	20.9-21.4	348	20.7	20.4-20.9	876	21.0	20.8-21.2
	Total	2,749	21.0†	20.9-21.3	2,098	20.5	20.3-20.7	4,847	20.8	20.7-21.0
North-China	18	2,665	21.4†	21.2-21.5	4,008	20.7	20.6-20.7	6,673	20.9	20.9-21.0
	19	1,679	21.5†	21.4-21.7	1,830	21.0	20.9-21.1	3,509	21.2	21.1-21.3
	20	949	22.0†	21.8-22.2	993	21.3	21.1-21.4	1,942	21.6	21.5-21.7
	Total	5,293	21.6†	21.4-21.7	6,831	20.9	20.8-21.0	12,124	21.1‡	21.1-21.2
East-China	18	3,022	20.0	19.9-20.1	5,187	20.1	20.0-20.2	8,209	20.1	20.0-20.1
	19	2,691	20.3	20.2-20.4	3,017	20.3	20.2-20.4	5,708	20.3	20.2-20.4
	20	2,018	20.7†	20.6-20.9	1,842	20.5	20.4-20.6	3,860	20.6	20.5-20.7
	Total	7731	20.2	20.1-20.4	10,046	20.2	20.1-20.3	17,777	20.3‡,¶	20.2-20.3
South-Middle	18	1,815	20.0†	19.9-20.1	4,489	19.5	19.4-19.5	6,304	19.6	19.6-19.7
	19	1,254	20.3†	20.2-20.4	2,154	19.7	19.6-19.8	3,408	19.9	19.9-20.0
	20	1,501	20.4†	20.3-20.5	1,376	19.9	19.8-20.0	2,877	20.1	19.9-20.2
	Total	4,570	20.2†	20.1-20.3	8,019	19.6	19.5-19.7	12,589	19.8‡,¶,§	19.8-19.9
North-West	18	395	20.4†	20.1-20.7	1,589	19.5	19.4-19.6	1,984	19.6	19.5-19.7
	19	271	20.4†	20.0-20.7	668	19.7	19.5-19.8	939	19.9	19.7-20.0
	20	243	20.9†	20.5-21.3	405	20.0	19.8-20.2	648	20.4	20.1-20.7
	Total	909	20.5†	20.2-20.8	2,662	19.7	19.5-19.8	3,571	19.9‡,¶,§	19.7-20.0
South-West	18	711	19.8†	19.7-20.0	3,250	19.6	19.5-19.7	3,961	19.6	19.6-19.7
	19	767	19.9	19.8-20.1	2,460	19.9	19.8-20.0	3,227	19.9	19.8-20.0
	20	646	20.2	20.0-20.4	2,031	20.1	20.0-20.2	2,677	20.1	20.0-20.2
	Total	2,124	19.9	19.8-20.1	7,741	19.8	19.7-19.9	9,865	19.8‡,¶,§	19.7-19.9
Whole Country	18	9,958	20.5†	20.4-20.6	19,683	19.9	19.9-20.0	29,641	20.1	20.1-20.2
	19	7,533	20.6†	20.6-20.7	10,719	20.2	20.1-20.2	18,252	20.4	20.3-20.4
	20	5,885	20.8†	20.8-20.9	6,995	20.3	20.3-20.4	12,880	20.5	20.4-20.6
	Total	23,376	20.6†	20.5-20.7	37,397	20.0	20.0-20.1	60,773	20.3	20.3-20.3

∗Comparison based on 95% CI of BMI;

†p<0.05 compared with rural areas;

‡p<0.05 compared with North-East;

¶p<0.05 compared with North-China;

§p<0.05 compared with East-China;

BMI=Body mass index; CI=Confidence interval

We could also see the differences in BMI between urban and rural residences in different geographical areas. The significant differences in BMI in the urban and rural areas were observed in each age-group of different areas, except for 18- and 19-year old groups in the East-China and 19- and 20-year old groups in the South-West areas. The difference between urban areas and rural areas was 0.6 for the whole country, with the largest difference (0.8) in the North-West. There were significant differences in BMI between urban areas and rural areas in the same areas, except for East-China and the South-West.

### Nutritional status of male youths of the Han nationality

The comparison of the nutritional status of male youths aged 18-20 years in different areas is shown in Table 2. The table shows that the prevalence of underweight in different age-groups decreased from 23.8% at the age of 18 years to 17.7% at the age of 20 years, with an average of 21.6%, while overweight tended to go higher from 4.1% at the age of 18 years to 5.6% at the age of 20 years, with an average of 4.6%. For each age-group, the prevalence of underweight in the North-West area was the highest (29.8%), with 33.3% for 18-year, 29.9% for 19-year, and 21.6% for 20-year old youths, while the prevalence of underweight in North-China was the lowest (14.3%), with 16.8% for 18-year, 13.4% for 19-year, and 9.8% for 20-year old youths. The prevalence of overweight and obesity in North-China was the highest (9.1% and 1.4% respectively), and the prevalence of overweight for each age-group was 8.0%, 8.9%, and 12.0% respectively, while the prevalence of obesity for each age-group was 1.4%, 1.5%, and 1.4% respectively. The prevalence of overweight in the South-Middle area was the lowest (2.2%), with 1.7% for 18-year, 2.4% for 19-year, and 3.2% for 20-year old youths. The prevalence of obesity in the South-West was the lowest (0.2%), with 0.1% for 18-year, 0.2% for 19-year, and 0.2% for 20-year old youths.

**Table 2:** Comparison of prevalence (%) of different nutritional status of male youths aged 18-20 years in different areas of China

Area	Age(years)	Urban areas∗				Rural areas				Total∗∗			
		Under-weight	Normal-weight	Over-weight	Obesity	Under-weight	Normal-weight	Over-weight	Obesity	Under-weight	Normal-weight	Over-weight	Obesity

North-East	18†	17.0	74.7	7.0	1.3	22.1	72.9	4.3	0.7	19.4	73.9	5.7	1.0
	19†	14.7	74.9	9.2	1.3	16.1	78.1	4.9	0.8	15.3	76.2	7.5	1.1
	20	11.6	80.7	6.8	0.9	11.8	84.2	3.4	0.6	11.6	82.1	5.5	0.8
	Total†	15.2	76.0	7.6	1.2	18.1	76.9	4.3	0.7	16.5	76.3	6.2	1.0
North-China	18†	15.8	70.9	10.8	2.4	17.4	75.7	6.1	0.7	16.8‡	73.8	8.0	1.4
	19†	13.6	72.9	11.1	2.4	13.2	79.3	6.8	0.7	13.4‡	76.3	8.9	1.5
	20†	9.1	74.3	14.4	2.2	10.6	79.2	9.6	0.7	9.8‡	76.8	12.0	1.4
	Total†	13.7	72.2	11.7	2.4	14.7	77.5	7.1	0.7	14.3‡	75.2	9.1	1.4
East-China	18†	26.1	69.1	4.4	0.4	22.3	74.2	3.3	0.3	23.7‡,¶	72.3	3.7	0.3
	19	23.8	69.7	5.7	0.8	19.2	76.9	3.6	0.3	21.4‡,¶	73.5	4.6	0.5
	20	19.4	72.3	7.4	0.9	16.7	78.9	4.1	0.3	18.1‡,¶	75.4	5.8	0.6
	Total	24.0	70.0	5.4	0.6	20.2	76.0	3.6	0.3	21.8‡,¶	73.3	4.4	0.4
South-Middle	18†	23.9	72.9	2.8	0.4	29.2	69.4	1.3	0.1	27.7‡,¶,§	70.4	1.7	0.2
	19†	20.4	74.9	4.1	0.6	24.7	73.7	1.4	0.1	23.2‡,¶,§	74.2	2.4	0.3
	20†	19.8	75.0	4.7	0.5	21.1	77.1	1.5	0.3	21.4‡,¶,§	76.0	3.2	0.4
	Total†	22.0	73.9	3.6	0.5	26.1	72.3	1.4	0.1	24.8‡,¶,§	72.7	2.2	0.3
North-West	18†	31.9	58.5	8.1	1.5	33.6	64.8	1.6	0.1	33.3‡,¶,§,††	63.5	2.9	0.4
	19	32.1	60.1	6.3	1.5	29.0	68.3	2.4	0.3	29.9‡,¶,§,††	65.9	3.5	0.6
	20	23.5	67.1	8.2	1.2	20.5	77.3	2.2	0.0	21.6‡,¶,§,††	73.5	4.5	0.5
	Total†	30.2	60.8	7.6	1.4	29.4	68.5	2.0	0.1	29.8‡,¶,§,††	66.3	3.4	0.5
South-West	18	31.4	63.7	4.5	0.4	27.3	71.2	1.4	0.1	28.0‡,¶,§,††	69.9	2.0	0.1
	19†	31.6	63.2	4.6	0.7	22.5	75.8	1.7	0.1	24.6‡,¶,§,††	72.8	2.4	0.2
	20	25.4	69.0	5.3	0.3	19.4	77.4	2.9	0.2	20.9‡,¶,§	75.4	3.5	0.2
	Total†	30.2	64.7	4.7	0.5	24.2	73.9	1.8	0.1	25.5‡,¶,§,††	71.9	2.4	0.2
Whole country	18†	22.3	70.2	6.3	1.1	24.6	72.1	3.0	0.3	23.8	71.5	4.1	0.6
	19†	21.0	70.9	6.9	1.2	20.5	76.0	3.3	0.3	20.7	73.8	4.8	0.7
	20†	17.9	73.5	7.6	1.0	17.5	78.3	3.9	0.3	17.7	76.1	5.6	0.6
	Total†	21.0	71.1	6.8	1.1	21.9	74.6	3.3	0.3	21.6	73.2	4.6	0.6

∗The statistical differences in nutritional status between urban and rural youths were tested by the Mann-Whitney U-test;

†p<0.05 compared with rural areas;

∗∗The statistical differences in nutritional status among different areas were tested by the Kruskal-Wallis test;

‡p<0.05 compared with North-East;

¶p<0.05 compared with North-China;

§p<0.05 compared with East-China;

††p<0.05 compared with Middle-South;

‡‡p<0.05 compared with North-West

The prevalence of underweight in urban areas was 21.0%, with 6.8% for overweight and 1.1% for obesity, and it was 21.9% in rural areas, with 3.3% for overweight and 0.3% for obesity. The prevalence of underweight in North-China was 13.7% in urban areas and 14.7% in rural areas, the lowest in China. However, the prevalence of overweight was 11.7% in urban areas and 7.1% in rural areas. The prevalence of obesity was 2.4% in urban areas and 0.7% in rural areas, the highest in China. The prevalence of underweight in the North-West was the highest—30.2% in urban areas and 29.4% in rural areas. The prevalence of overweight in the South-Middle areas was the lowest; it was 2.2% in the whole areas, with 3.6% in urban areas and 1.4% in rural areas. The prevalence of obesity in the South-West area was the lowest; it was 0.2% in the whole areas, with 0.5% in urban areas and 0.1% in rural areas.

## DISCUSSION

Geographic differences in the nutritional status of children and adolescents exist in most countries (7,9,10). Buttriss reported that BMI was significantly associated with geographical regions: boys in the South-East England and London region had a lower average BMI than boys in other regions, and BMI of boys from Scotland was higher than average (10). This study found that the nutritional status of male youths of the Han nationality in different areas of China varied greatly. Compared to other areas, male youths in the Northern regions of China had a higher BMI, higher prevalence of overweight and obesity, and a lower prevalence of underweight, while male youths in the Western regions had a smaller BMI, a higher prevalence of underweight, and lower prevalence of overweight and obesity than those in other areas. In addition, significant differences in BMI and different prevalence of different nutritional status exist in urban and rural areas of China, with a higher BMI, higher prevalence of overweight and obesity, and a lower prevalence of underweight among urban male youths than among rural male youths.

It has been generally recognized that body-weight and the prevalence of overweight and obesity are related to socioeconomic factors. Obert *et al.* reported that the risk of being overweight was more related to geography than to demographic variables, such as income and family background (11). Since China is a developing country, its economic status and development speed are quite different in different areas. In 2001, the gross domestic product (GDP) per capita of the North-West and South-West regions was US$ 704.5 and US$ 599.3 respectively, the lowest among the six areas, with US$ 1,828.4 in East-China and US$ 1,518.5 in North-China (12), the highest among the six areas. In 1999, pure percapita income was US$ 731.8 in urban areas and US$ 276.3 in rural areas, the ratio of consumption between urban areas and rural areas was 1:3.4, and the ratio of total consumption between urban areas and rural areas was 70:30, whereas the ratio of population between urban areas and rural areas was 30:70 (12)^.^ Undoubtedly, all these remarkable differences in socioeconomics have deeply influenced the living standards and styles of thousands of Chinese households and, hence, affected the nutritional status of youths. It is well-known that socioeconomic status, family, and food supply are among the most important environmental factors that affect the nutritional status of youths. The difference in economic status and development speed among different areas could explain the difference in nutritional status.

The co-existence of undernutrition and overnutrition has been experienced recently by many developing countries of the world (9,13–15). This study found that the co-existence also occurred in China, with underweight being slightly more prevalent. Although overweight was emerging as a problem in some areas, the prevalence was still quite low compared to other developed countries. The above results suggest that, although Chinese youths have experienced an improvement in diet and nutritional status, underweight is still an important nutritional problem, especially among youths in the Western regions of China. The co-existence of underweight and overweight in China can also be explained by the difference in economic status and development speed among different areas.

Since 1975, several large national anthropometric surveys of people of different ages were carried out in China. Six national constitution and health surveys of students aged 7-22 years were conducted in 1979, 1985, 1991, 1995, 2000, and 2005 (16,17), three national growth and development surveys of children aged 0-7 year(s) were conducted in 1975, 1985, and 1995, and two national constitution surveys of people aged 3-69 years were conducted in 2000 and 2005 (18). In addition, the National Fitness Supervising and Testing Network of Students aged 7-22 years was established in 2002 (19). Results of these surveys are important to learn the status and trends in the change of health of the Chinese people. However, different institutions organized different surveys, and the sampling-spots and the survey and evaluation methods were not consistent, so the data of different surveys usually cannot be shared, and only the same survey in different years can be compared. Results of all these surveys have the same conclusion: the observed rates of obesity and overweight for different age-groups have been increasing year by year and decreasing for underweight (16–19). For example, in 2005, the detection rates of underweight for urban boys, urban girls, rural boys, and rural girls aged 7-22 years were 21.61%, 32.74%, 25.79 %, and 34.15% respectively, whereas these decreased by 2.1%, 1.5%, 1.3%, and 1.3% respectively compared to those in 2000. The detection rates of overweight for urban boys, urban girls, rural boys, and rural girls aged 7-22 years were 13.25%, 8.72%, 8.20%, and 4.61% respectively, and the increasing rates were 1.4%, 0.7%, 1.8%, and 1.2% respectively compared to those in 2000. The detection rates of obesity for urban boys, urban girls, rural boys, and rural girls aged 7-22 years were 11.39%, 5.01%, 5.07%, and 2.63% respectively, and the increasing rates were 2.7%, 0.9%, 1.6%, and 0.4% respectively compared to those in 2000 (16).

Since military service is compulsory in China, all male youths aged 18 years have the obligation to serve in the army; so, the physique status of recruited male youths can reflect the physical status of male youths in China. Of the 56 nationalities in China, the Han is the biggest nationality, and its population occupies 91.02% of the whole country (12). It is, thus, critical to study the nutritional status of male youths of the Han nationality for improving their nutritional status.

It can be concluded that nutritional status is the result of multiple factors working together. Our results suggest that there are dramatic differences in the nutritional status in different areas. Underweight is still prevalent among all Chinese male youths. Therefore, we should pay more attention not only to the prevention of overweight, especially for North-China, but also to that of the high proportion of underweight among young men, especially in the Western areas.
